# Impact of Heat Treatment and Building Direction on Selective Laser-Melted 316L Stainless Steel: Microstructure and Mechanical Properties

**DOI:** 10.3390/ma18010032

**Published:** 2024-12-25

**Authors:** Zequn Feng, Haijun Pan, Wangping Wu, Zhizhi Wang, Peng Jiang

**Affiliations:** School of Mechanical Engineering and Rail Transit, Changzhou University, Changzhou 213164, China; s22050802002@smail.cczu.edu.cn (Z.F.); phj@cczu.edu.cn (H.P.); wuwping@cczu.edu.cn (W.W.)

**Keywords:** selective laser melting, 316L steel, microstructure, anisotropy, heat treatment

## Abstract

Variations in the microstructural morphology with building direction during selective laser melting (SLM) result in the anisotropic mechanical properties of the specimens, while heat treatment effectively reduces this anisotropy. The degree of anisotropy of the material can be assessed by calculating the variance (σ) of the mechanical properties (strength, hardness) at different building directions at different temperatures. In this work, the effects of heat treatment temperatures (450°, 750 °C, and 1050 °C) and building directions (0°, 45°, 60°, and 90°) on the microstructure, hardness, and tensile properties of selective laser melting (SLM) SS316L were investigated. Unheated SLM SS316L specimens exhibit significant anisotropy (σ_UTS_ = 16.67, σ_UE_ = 9.60, and σ_HV_ = 9.60), while heat treatment effectively reduces this anisotropy. As the heat treatment temperature increases, the melt pool boundaries disappear and grains become uniform at 750 °C, significantly reducing the anisotropy of the mechanical properties (σ_UTS_ = 14.65, σ_UE_ = 4.33, σ_HV_ = 6.72). At 1050 °C, the grain size increases slightly, resulting in a minor rise in anisotropy (σ_UTS_ = 14.93, σ_UE_ = 4.97, σ_HV_ = 8.39) compared to that seen at 750 °C. After heat treatment, the SLM SS316L specimen heated at 450 °C shows the lowest anisotropy. Notably, the specimens built in the 0° direction and heated at 450 °C exhibit finer sub-grains and enhanced mechanical properties, achieving a hardness of 244.43 HV, a tensile strength of 655.85 MPa, and an elongation of 21.25%.

## 1. Introduction

Selective laser melting (SLM), a significant branch of laser powder bed fusion (L-PBF), operates on the principle of selectively melting metal powder using a laser beam. During processing, the laser scans the metal powder layer according to the preset CAD data path, melting the powder and building it layer by layer to form a three-dimensional solid [1,2]. SLM has notable advantages in forming complex internal structures compared to traditional forging and casting. It eliminates the need for complex mold designs, allowing precise parts to be produced directly from design data. Additionally, SLM-fabricated parts typically require minimal steps post-processing, effectively reducing production costs and shortening lead times [3,4].

On balance, 316L stainless steel (SS316L) is widely used in marine, medical, and aerospace fields due to its good plasticity, high-temperature resistance, and corrosion resistance [5,6,7]. Compared to conventional manufacturing methods, SLM-produced SS316L has a finer microstructure (several microns), while conventionally manufactured SS316L has hundreds of microns. This results in SLM SS316L having significantly higher tensile strength than conventional SS316L [8]. However, due to the extremely high cooling rates of the SLM process (10^3^ to 10^8^ K/s) [9], pores and high residual stress are present in the formed parts [10]. To address these issues, many studies have optimized SLM process parameters, such as scanning speed, scanning angle, and laser power density, to improve the material’s performance [11,12,13,14,15]. Although these optimizations significantly increase strength, the elongation is usually below 13% [1,16,17].

Additionally, SLM parts exhibit anisotropy in mechanical properties and microstructures between vertical (z direction) and horizontal (x and y directions) building directions. This is due to the thermal gradient differences present in the vertical and horizontal directions [8,18,19,20]. To address this issue, researchers have applied various heat treatment processes. Tomus et al. [21] conducted annealing at 1175 °C for 2 h and hot isostatic pressing (HIP) at 1175 °C/150 MPa for 2 h on SLM-formed Hastelloy X, significantly reducing anisotropy, with tensile strengths in the deposition and scanning directions becoming nearly identical. Takaichi et al. [22] studied the heat treatment of SLM-formed Co-Cr-Mo alloy and found that heating at a rate of 60 °C/min to 1150 °C, holding for 6 h, and then furnace-cooling to 300 °C transformed columnar grains into equiaxed grains, markedly reducing texture and improving anisotropy in mechanical properties. Divya [23] and Sanchez [24] used high-temperature annealing to mitigate microstructural anisotropy, balancing strength and ductility and thus optimizing overall performance. Vialro et al. [20] improved the anisotropy of SLM-formed Ti6Al4V alloys through high-temperature aging treatment.

While significant research has focused on the process parameters and mechanical properties of SLM 316L, the effect of build direction on its performance has not been thoroughly studied. Existing studies mainly examine horizontal, vertical, and 45° directions; this work extends the analysis to include the 60° direction. Additionally, most research has concentrated on optimizing process parameters and heat treatment, with limited investigation into the combined effects of build direction and heat treatment on microstructure and properties. Therefore, this work analyzes the microstructures of SLM SS316L specimens with various forming directions (0°, 45°, 60°, and 90°), and compares the microstructure and tensile properties of specimens before and after heat treatment at different temperatures. By observing the microstructure and fracture morphology of specimens under different heat treatment temperatures and forming directions using Optical Microscopy (OM) and Scanning Electron Microscopy (SEM), this work will provide technical insights to allow for the control of the microstructure and properties of the SLM SS316L.

## 2. Experiment

### 2.1. Materials and Preparation

**Figure 1:**
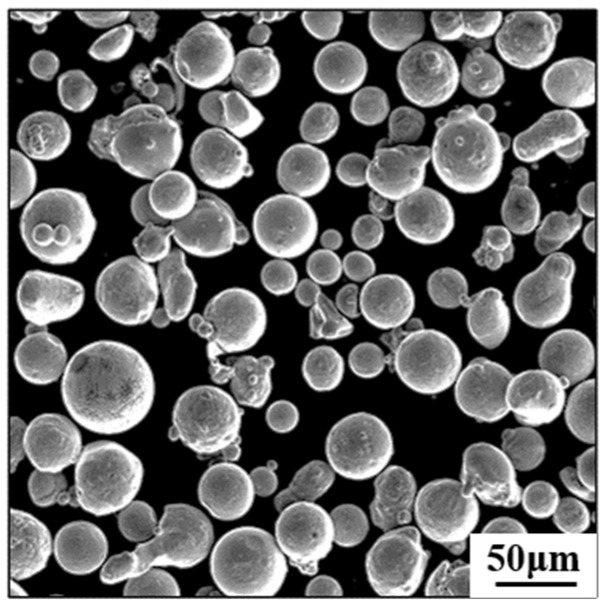
SEM image of powder particle morphology.

The material used in this experiment is gas-atomized SS316L powder, and its chemical composition is presented in Table 1. Figure 1 shows the spherical morphology of SS316L; the SS316L powder particles primarily exhibit spherical or ellipsoidal shapes, with an average size ranging from 21 to 53 μm. Local agglomeration is observed.

**Figure 2:**
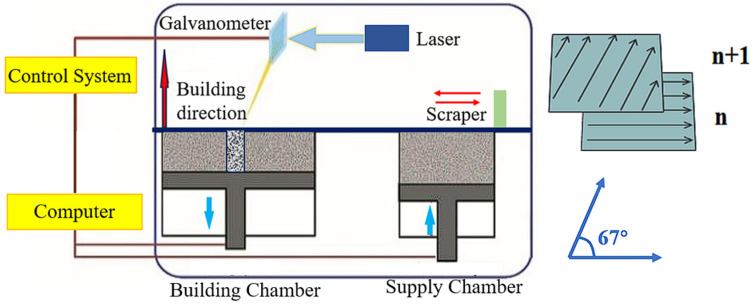
The schematic diagram of the preparation process of SLM SS316L.

Figure 2 shows the schematic diagram of the preparation process of SLM 316L specimens. The SLM SS316L specimens were fabricated using an ISLM160 device (Z Rapid Tech. Co., Ltd., Suzhou, China). The specific parameters for preparing SLM SS316L were a laser power of 200 W, a scanning speed of 1000 mm/s, a layer thickness of 0.03 mm, and a scanning direction of 67°.

**Figure 3:**
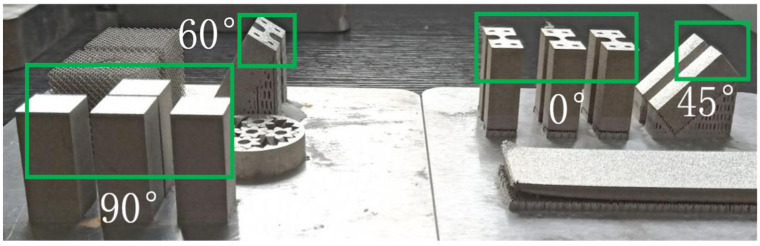
Different building directions of SLM SS316L manufactured by SLM.

**Figure 4:**
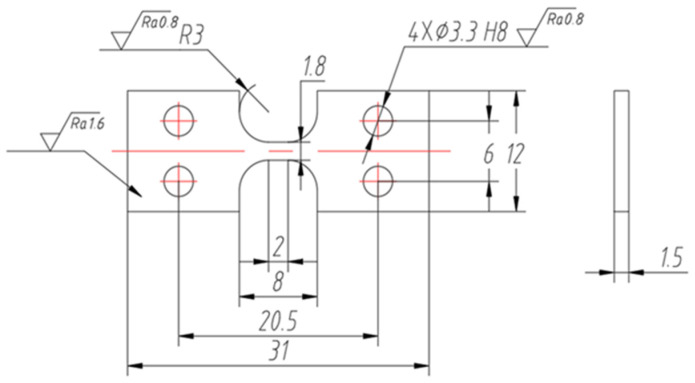
The dimensions of the tensile specimens.

The specimens with building directions of 0°, 45°, 60°, and 90° were constructed according to the parameters, as shown in Figure 3. Due to the existence of an overhanging structure [25] in the 90°-formed specimens, to ensure the compactness of the specimens, cubic specimens were constructed with dimensions of 8 mm × 8 mm × 3 mm, and then tensile portions were cut using Wire Electrical Discharge Machining (WEDM) cutting (Sanguang Science & Technology Co., Ltd., Suzhou, China). Figure 4 shows the dimensions of the tensile specimens.

### 2.2. Heat Treatment

To examine the influence of heat treatment (HT) temperature on the microstructure and properties of SLM SS316L, specimens built at different angles (0°, 45°, 60°, 90°) were subjected to heat treatment, as outlined in Table 2. For instance, the HT450-45 specimen refers to an SLM SS316L sample fabricated at a 45° angle relative to the horizontal plane and heat-treated at 450 °C. The heat treatment process was carried out in a LFM1400C furnace (Compazine Equipment Technology Co., Ltd., Hefei, China), with a heating rate of 10 °C/min. Specimens without heat treatment (UHT) were used for comparison.

### 2.3. Microstructural Characterization 

The fabricated specimens were cut into 10 mm × 10 mm × 5 mm pieces and ground successively using abrasive papers with grit sizes of 400#, 800#, 1000#, 1500#, and 2000# to achieve smoothness. Subsequently, the specimen was electrolytically polished using a 5% (by volume) perchloric acid alcohol solution at 30 V for 15 s. After polishing, the specimens were rinsed with clean water, air-dried, and then etched with aqua regia (a 3:1 mixture of concentrated hydrochloric acid and nitric acid). The microstructures of the specimens were observed using OM and SEM. Phase analysis of the specimens before and after heat treatment was conducted using a D/Max-2500 X-Ray Diffractometer (XRD, Rigaku Corporation, Akishima-shi, Japan), with a scanning range from 30° to 100° and a step size of 0.02°.

### 2.4. Properties Tests

The hardness of SLM SS316L specimens was assessed using an HVS-1000 hardness tester (Aolong Xingdi Testing Equipment Co., Ltd., Shanghai, China), with a test force of 1 N applied for 10 s. Nine random points were measured on each sample to enhance experimental accuracy, and the average value was calculated. Tensile tests were performed on the SLM SS316L specimens using an AGS-10KND precision universal testing machine (Hensgrand Instrument Co., Ltd., Jinan, China), operating at a tensile speed of 1 mm/min. Following the tensile tests, the fracture surface morphology of the broken specimens was examined using SEM.

## 3. Result and Discussion

### 3.1. Microstructure

**Figure 5:**
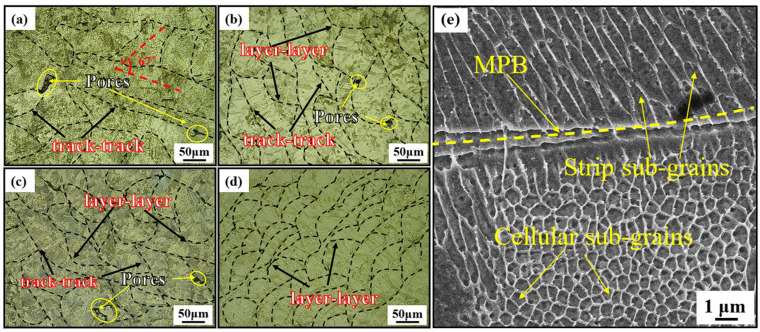
Images of the UHT specimens in (**a**) 0°, (**b**) 45°, (**c**) 60°, and (**d**) 90° building directions, and (**e**) the high-magnification microstructure within the molten pool.

Figure 5 shows the microstructures of the UHT specimens built in four directions. Irregular pores caused by fusion defects are commonly found in the SLM specimens, as extensively discussed in the literature [26,27,28]. The microstructures of the specimens formed by SLM through the melting of metal powder exhibit two types of melt pool boundaries (MPBs): layer–layer and track–track [29]. Specifically, “layer–layer” refers to the interface between successive layers of material, while “track–track” refers to the interface between individual laser tracks within a single layer. Figure 5a displays the microstructure of the UHT-0 specimen. The MPBs are marked by black dashed lines, with the track–track MPBs in the 0° direction forming an angle of approximately 67°, consistent with the scanning directions. Figure 5b displays the microstructure of the UHT-45 specimen, showing the MBPs primarily composed of layer–layer MPBs with fewer track–track MPBs. The average width of the track–track melt pools (MPs) is 67.8 μm. Figure 5c shows the microstructure of the UHT-60 specimen. Compared to the UHT-45 specimen, the UHT-60 specimen exhibits both layer–layer and track–track MPBs, with an increased proportion of layer–layer overlap. Moreover, the average width of the track–track MPs decreases to 59.4 μm. Figure 5d displays the microstructure of the UHT-90 specimen, featuring tightly overlapped and fish-scale layer–layer MPBs. The microstructure within the MPs of the UHT-90 specimen was further examined using SEM (Figure 5e), revealing finer sub-grain structures (columnar and cellular sub-grains). The average cell sub-grain size is 0.604 μm. This results from irregular energy output during the SLM process, leading to the formation of a special microstructure.

**Figure 6:**
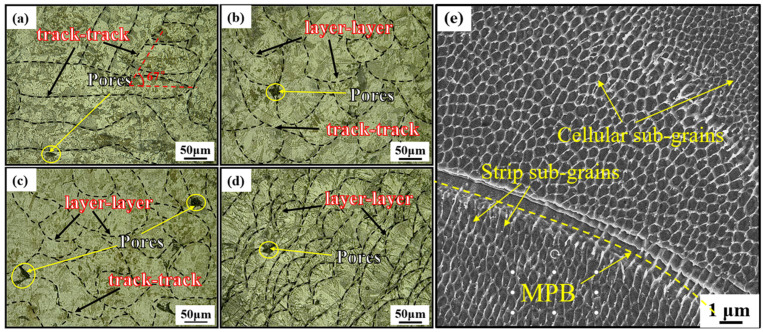
Images of the HT450 specimens in (**a**) 0°, (**b**) 45°, (**c**) 60°, and (**d**) 90° building directions, and (**e**) the high-magnification microstructure within the molten pool.

The MPBs (indicated by black dashed lines) of HT450 specimens are similar to those of UHT specimens, as shown in Figure 6a–d. SEM images (Figure 6e) of the interior of the MPs reveal columnar and cellular sub-grains in the HT450-90 specimen, with an average cellular sub-grain size of 0.533 μm. Compared to UHT, the sub-grains in HT450 are more refined.

**Figure 7:**
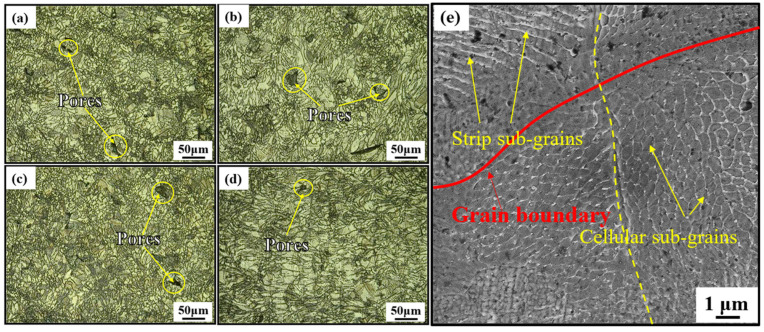
Images of the HT750 specimens in (**a**) 0°, (**b**) 45°, (**c**) 60°, an (**d**) 90° building directions, and (**e**) the high-magnification microstructure within the molten pool.

Figure 7a–d shows the microstructures of the HT750 specimens built in four directions. Compared to UHT and HT450 specimens, the MPBs of the HT750 specimens are decomposed due to the growth of grains and sub-grains, which leads to the disappearance of MPBs [30]. Moreover, the grains tend to become equiaxed and uniform. SEM observations of the HT750-90 specimen reveal the disappearance of some columnar and cellular sub-grains in the grains, with an average cellular sub-grain size of 0.635 μm, as shown in Figure 7e.

**Figure 8:**
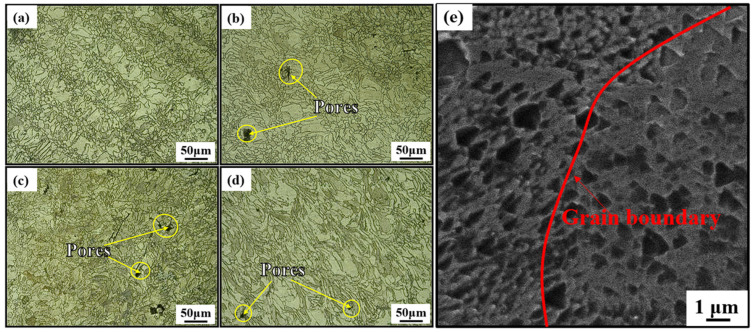
Images of the HT1050 specimens in (**a**) 0°, (**b**) 45°, (**c**) 60°, and (**d**) 90° building directions, and (**e**) the high-magnification microstructure within the molten pool.

Figure 8a–d shows the microstructure of the HT1050 specimens built in four directions. The grains grew larger, and the grain uniformity in different building directions decreased. As shown in Figure 8e, the columnar and cellular sub-grains grew further to form austenite grains in the HT1050-90 specimen.

**Figure 9:**
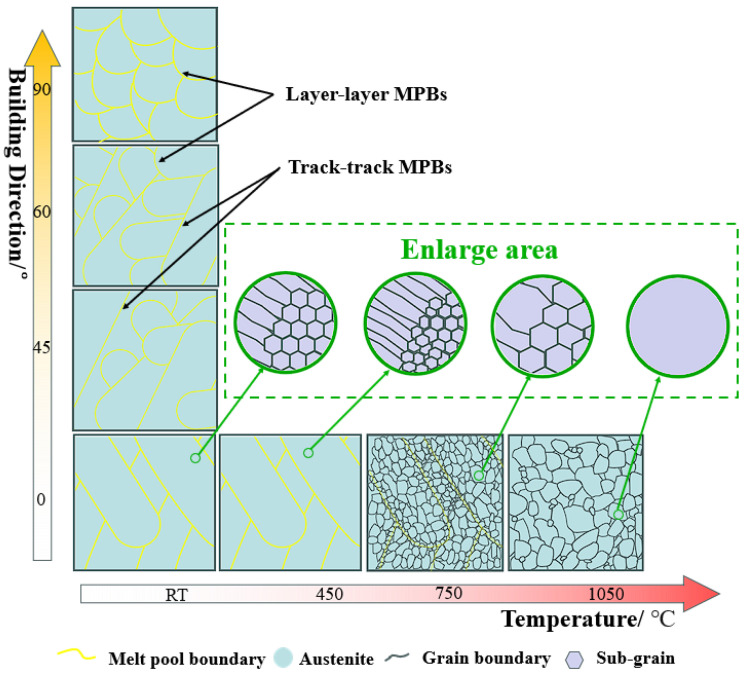
A schematic of the microstructure of SLM SS316L at different building directions and 0° after heat treatment (with insets showing magnified sub-grain changes in the molten pool or austenite).

As the building direction increases from 0° to 90°, the overlap pattern of the molten pools changes from track-by-track to layer-by-layer due to the similar trends in microstructural changes observed in specimens at different building directions under the same heat treatment conditions (MPBs disappear → austenite grains form → sub-grains merge and austenite grains grow). Thus, the schematic depicting the microstructural evolution of SLM SS316L at different building directions during heat treatment can be representative of the changes observed at the 0° direction, as shown in Figure 9.

**Figure 10:**
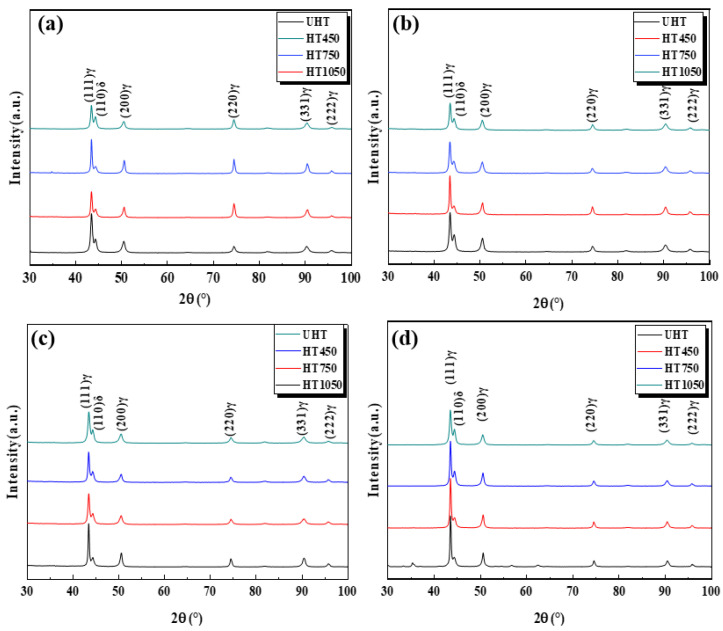
The XRD patterns of four annealed SLM SS316L specimens at different building directions. (**a**) 0°; (**b**) 45°; (**c**) 60°; (**d**) 90°.

Figure 10 presents the XRD patterns of the UHT, HT450, HT750, and HT1050 specimens in different building directions. The heated SLM SS316L consists of an austenite phase (γ phase), with a small amount of ferrite (δ phase). To calculate the degree of preferred orientation of different crystal planes, the Relative Texture Coefficient—*RTC*(*hkl*)—was introduced, as defined in Equation (1).

(1) $$RTC(hkl)=\frac{\frac{I(hkl)}{I_{0}(hkl)}}{\sum_{n}\frac{I(hkl)}{I_{0}(hkl)/n}}\times 100\%$$

where *I*(*hkl*) and *I*_0_(*hkl*) represent the measured intensity and the standard intensity from the JCPDS database of the (*hkl*) crystal plane, respectively. The variable *n* denotes the number of diffraction peaks. When *RTC*(*hkl*) > 1, it indicates that the preferred orientation occurs in the (*hkl*) crystal plane.

Table 3 presents the *RTC* values of the UHT, HT450, HT750, and HT1050 specimens for the (111)γ, (200)γ, (220)γ, (331)γ, and (222)γ crystal planes. The (111) and (222) crystal planes exhibit a certain preferential orientation. Additionally, the *RTC* value of the HT specimens decreases compared to the UHT specimens for the (111) plane and increases for the (222) plane.

### 3.2. Hardness

**Figure 11:**
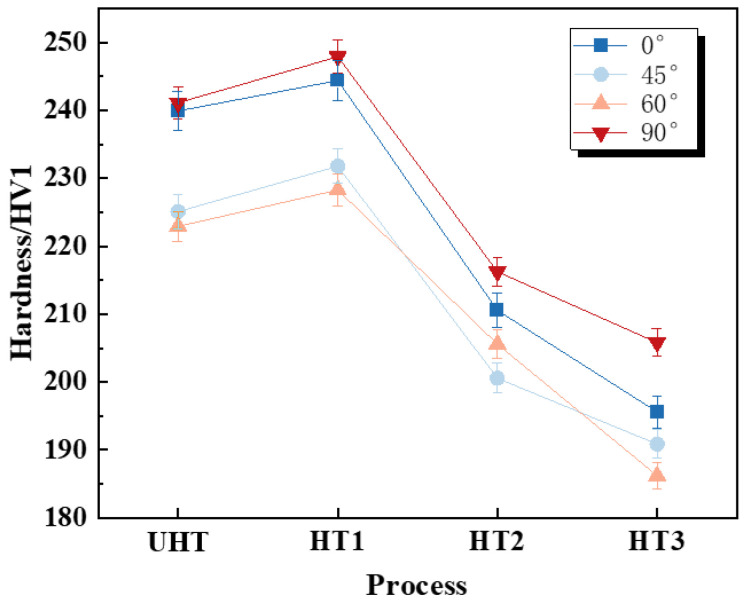
The hardness variations in SLM SS316L at different heat treatment temperatures.

Figure 11 shows the hardness variations in the UHT, HT450, HT750 and HT1050 specimens, with the corresponding data presented in Table 4. The hardness of the UHT specimens initially increases and then decreases with rising heat treatment temperatures. At 0° and 90°, the melting and bonding of metal powder between layer-to-layer and track-to-track overlap produces tighter particle connections and more uniform grain developments, enhancing the material’s hardness. As the forming angle increases, the melting and bonding of the metal powder are affected by an oblique influence, causing the interlayer connections to suffer from incomplete melting or crystallization, which leads to reduced hardness. Additionally, from 45° to 90°, the increase in sample angle and the number of layers leads to a tendency for residual stress to increase [31]. This causes grain boundaries to shift and twist, resulting in a smaller grain size and an increased number of grain boundaries, thereby enhancing the material’s hardness.

The average hardness of the UHT specimens is 232.3 HV. The HT450 specimen exhibits the highest average hardness of 236.6 HV, representing a 1.9% increase compared to the UHT specimen, and the hardness of the HT750 and HT1050 gradually decreases, showing reductions of 11.4% (208.2 HV) and 16.3% (194.6 HV), respectively. The hardness of SLM SS316L is mainly influenced by its microstructure and internal stresses. HT450 specimen refines the internal grains of the MBs, increasing the hardness. As the heat treatment temperature rises and goes beyond the recrystallization temperature (900–950 °C) of SS316L, grain coarsening occurs, leading to a decrease in hardness. As the heat treatment temperature increases, the dislocation density reduces [30], leading to a decrease in hardness. Table 4 presents the standard deviations (σ) and the ratio of the mechanical properties (UTS and EL) at different building directions (45°, 60°, and 90°) relative to the 0° direction. As shown in Table 4, below the recrystallization temperature, HT reduces the material’s degree of anisotropy. The specimens treated at 750 °C exhibit the lowest degree of anisotropy in elongation. However, above the recrystallization temperature, grain growth leads to reduced uniformity in grain size for the four different building directions compared to that of specimens heated at 750 °C, thereby increasing the degree of elongation anisotropy.

### 3.3. Tensile Properties

**Figure 12:**
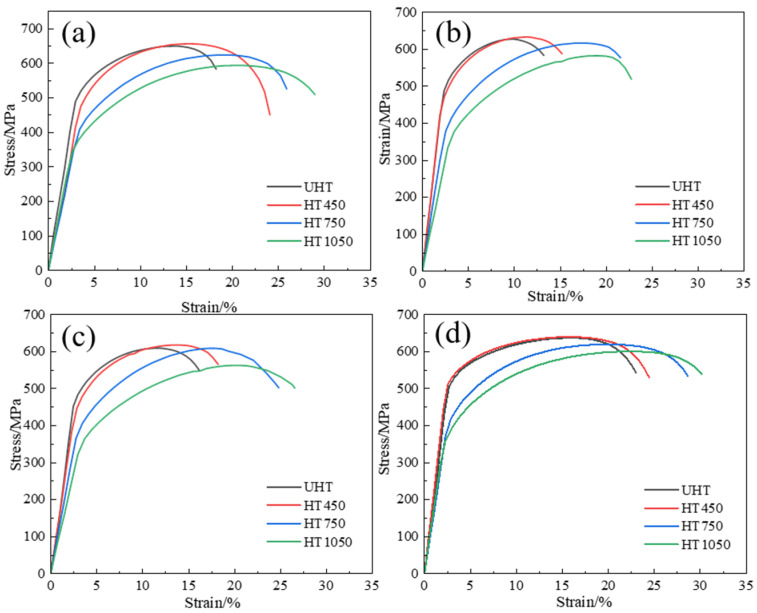
The tensile curves of SLM SS316L for UHT and HTs specimens (**a**) 0°, (**b**) 45°, (**c**) 60°, and (**d**) 90°.

**Figure 13:**
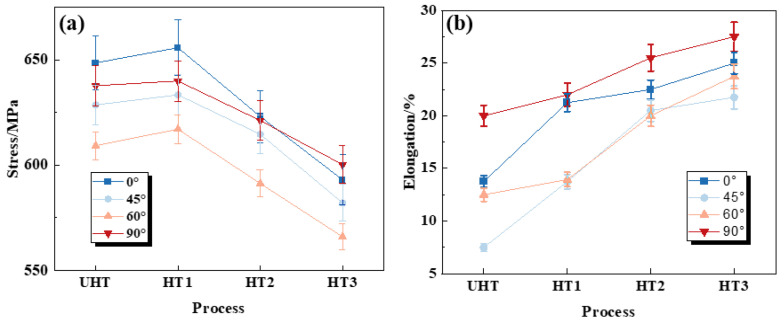
The tensile properties of SLM SS316L for UHT and HTs specimens (**a**) UTS and (**b**) EL.

The tensile curves of SLM SS316L for UHT and HTs specimens are shown in Figure 12, and Figure 13 presents the Ultimate Tensile Strength (UTS) and Elongation (EL) corresponding to the stress–strain data of all specimens, as shown in Table 5. The UTS values of UHT-0, UHT-45, UHT-60, and UHT-90 specimens are 637.61 MPa, 637.61 MPa, 608.13 MPa, and 583.03 MPa, respectively; the EL values are 13.75%, 7.5%, 12.5%, and 20%, respectively. These results demonstrate significant anisotropy in tensile properties. As shown in Table 5, as the heat treatment temperature increases, the anisotropy of the SLM SS316L specimens gradually decreases, reaching its lowest point at 750 °C. At 1050 °C, the anisotropy slightly increases. In contrast to UHT, the UTS of HT 1, HT 2, and HT 3 decreased with increasing heat treatment temperature, but the plasticity was significantly increased, as shown in Table 5. Notably, the UTS and EL values of samples built in the 0° and 90° directions are generally higher than those built in the 45° and 60° directions.

In the SLM-forming process, the higher tensile properties in the horizontal and vertical directions compared to 45° and 60° are primarily due to the uniform melt pool morphology, which reduces stress concentration. According to the literature [32], the mechanical properties of SLM SS316L are determined by the presence of cellular crystals rather than columnar crystals. In the HT450 specimens, grain refinement occurs and there is an increase in grain boundaries, which hinders the movement and diffusion of dislocations, leading to dislocation pile-up and increased material strength [33]. However, as the heat treatment temperature increases, grains grow larger. According to the Hall–Petch relationship, the tensile strength decreases. The melt pool influences the elongation of SLM parts. In the HT750 and HT1050 specimens, the MBPs disappear, reducing deformation resistance, lowering strength, and increasing toughness [34].

**Figure 14:**
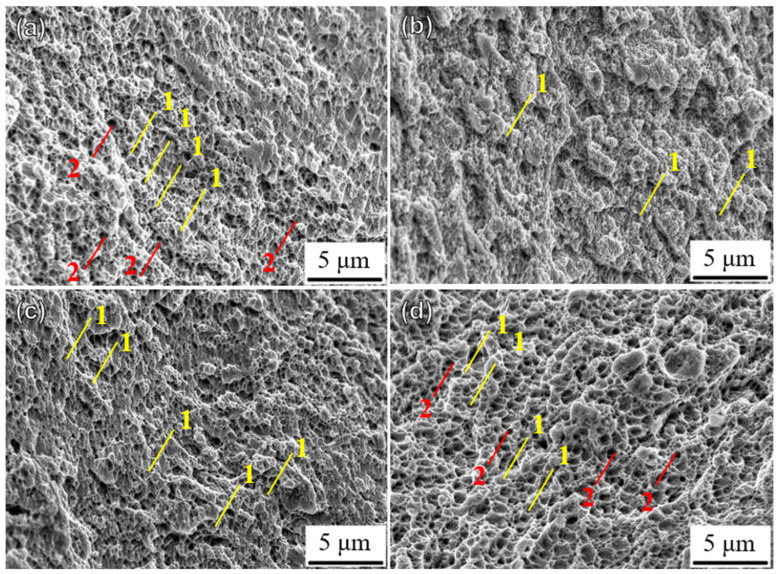
Fracture morphology of unheated SLM SS316L in (**a**) 0°, (**b**) 45°, (**c**) 60°, and (**d**) 90° building directions: 1—smaller, shallower dimples; 2—larger, deeper dimples.

Figure 14 shows the microscopic fracture morphologies of the UHT specimens built in four directions. The fracture morphologies of the UHT-90 specimen exhibit larger and deeper dimples, while the UHT-0 specimen shows smaller and shallower dimples. Compared to the UHT-0 and UHT-90 specimens, the UHT-45 and UHT-60 specimens have less noticeable dimples.

**Figure 15:**
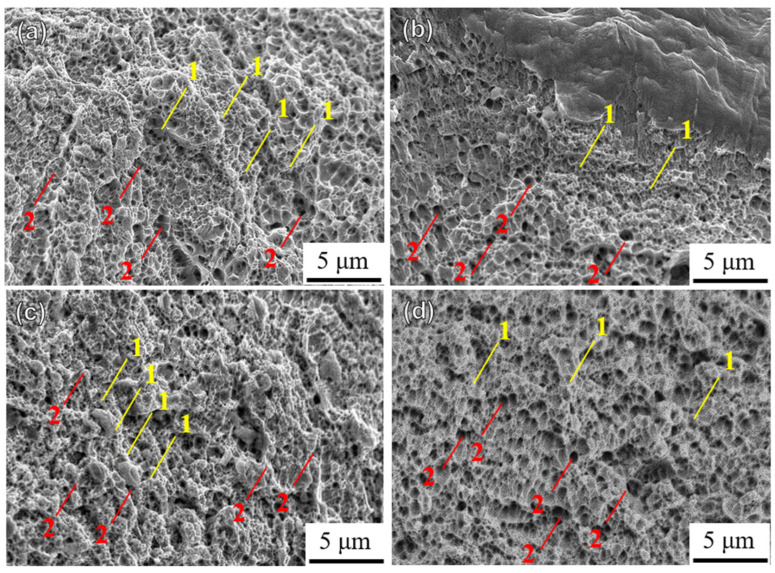
The fracture morphology of 450 °C heated SLM SS316L in (**a**) 0°, (**b**) 45°, (**c**) 60°, and (**d**) 90° building directions: 1—smaller, shallower dimples; 2—larger, deeper dimples.

Figure 15 shows the microscopic fracture morphologies of the HT450 specimens built in four directions. Deep and shallow dimples are visible in the HT450-0 and HT450-90 specimens (Figure 15a,d), with larger dimples in the 90° direction, corresponding to higher elongation. Compared to the UHT-45 and UHT-60 samples, the HT450-45 and HT450-60 samples show fewer small and dense dimples (Figure 15b,c).

**Figure 16:**
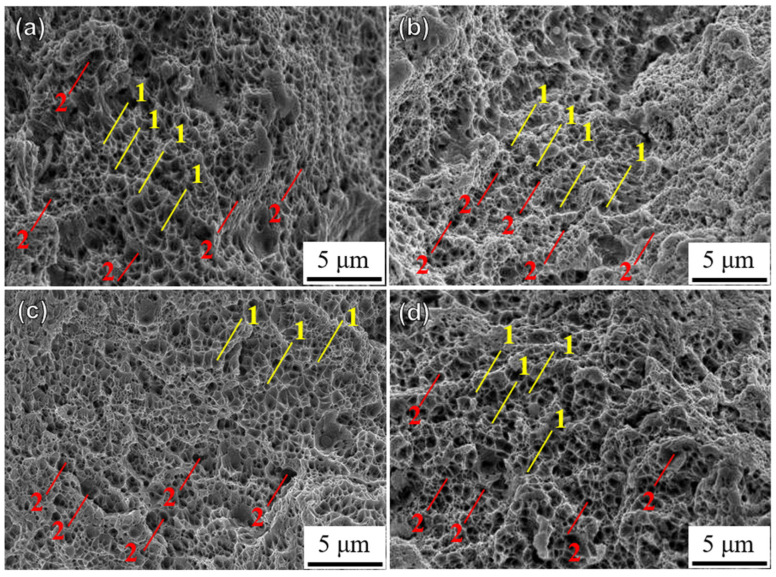
The fracture morphology of 750 °C heated SLM SS316L in (**a**) 0°, (**b**) 45°, (**c**) 60°, and (**d**) 90° building directions: 1—smaller, shallower dimples; 2—larger, deeper dimples.

**Figure 17:**
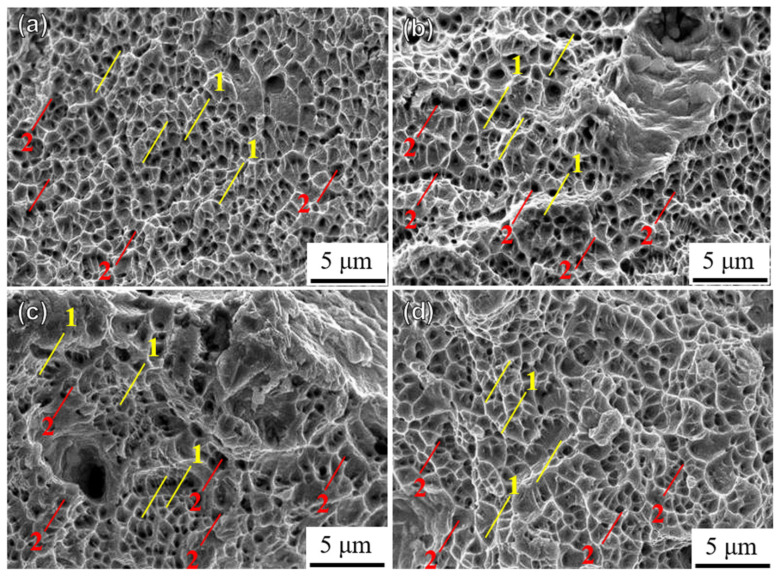
The fracture morphology of 1050 °C heated SLM SS316L in (**a**) 0°, (**b**) 45°, (**c**) 60°, and (**d**) 90° building directions: 1—smaller, shallower dimples; 2—larger, deeper dimples.

Figure 16 shows the microscopic fracture morphologies of the HT750 specimens built in four directions. Compared to the HT450 specimens, HT750 fractures are more uniform, greatly reducing the anisotropy of the tensile properties. The dimples in the HT1050 specimens are significantly larger, and the fracture surface consists mainly of equiaxed dimples (Figure 17). Due to the presence of voids in the HT1050-45 and HT1050-60 specimens, the stress distribution is uneven, causing the voids to coalesce into larger dimples on the fracture surface, slightly increasing the anisotropy of the tensile properties.

## 4. Conclusions

This study attempted to understand the influence of building directions and heat treatment on the mechanical properties of SLM TC4. From this research, the following conclusions can be drawn:

The microstructures of the specimens built in 0° direction show track–track melt pool boundaries (MPBs), while in the 90° direction, there are layer–layer MPBs. In the 45° and 60° directions, the microstructures show both types of MPBs. After heating at 450 °C, the morphology of molten pools (MPs) did not change greatly. However, the sub-grains inside the molten pool are refined. At 750 °C, the MPBs begin to decompose, and grains start to coarsen. Some of the sub-grains inside the MPs become unclear. After heating at 1050 °C, the grains grow, and the MPBs disappear together with the sub-grains.

The tensile properties of SLM SS316L exhibit significant anisotropy. As the heat treatment temperature increases, the anisotropy gradually decreases, slightly increasing at 1050 °C but remaining lower than that of unheated specimens. The unheated specimens built in the 0° direction show the best overall performance, with a hardness of 239.93 HV, a tensile strength of 648.61 MPa, and an elongation of 14.51%. In all heated specimens, HT450-0 specimens exhibit the best overall mechanical properties, with a hardness of 244.43 HV, a tensile strength of 655.85 MPa, and an elongation of 21.25%. Notably, when the heat treatment temperature was increased, the elongation of the SLM SS316L sample greatly improved in all directions.

## Figures and Tables

**Table 1:** Chemical composition of SS316L powder.

Element	Si	Mn	P	S	Cr	Ni	Mo	N	O	Fe
Component/%	0.470	0.850	0.034	0.005	16.340	10.380	2.130	0.034	0.003	Balance

**Table 2:** The heat treatment scheme of SLM 316L (AC = air cold).

Specimen	Building Direction/°	Procedure
UHT-0	0	-
UHT-45	45	-
UHT-60	60	-
UHT-90	90	-
HT450-0	0	450 °C/1.5 h/AC
HT450-45	45	450 °C/1.5 h/AC
HT450-60	60	450 °C/1.5 h/AC
HT450-90	90	450 °C/1.5 h/AC
HT750-0	0	750 °C/1.5 h/AC
HT750-45	45	750 °C/1.5 h/AC
HT750-60	60	750 °C/1.5 h/AC
HT750-90	90	750 °C/1.5 h/AC
HT1050-0	0	1050 °C/1.5 h/AC
HT1050-45	45	1050 °C/1.5 h/AC
HT1050-60	60	1050 °C/1.5 h/AC
HT1050-90	90	1050 °C/1.5 h/AC

**Table 3:** The *RTC* values of the UHT, HT450, HT750, and HT1050 specimens for the (111)α, (200)α, (220)α, (331)α, and (222)α crystal planes.

Specimen	*RTC* (111)	*RTC* (200)	*RTC* (220)	*RTC* (331)	*RTC* (222)
UHT-0	1.7925	0.7845	0.25	0.6175	1.5555
HT450-0	1.318	0.794	0.5745	0.8165	1.4965
HT750-0	1.3595	0.768	0.4595	0.8	1.6135
HT1050-0	1.4715	0.684	0.481	0.776	1.588
UHT-45	1.6435	0.852	0.192	0.61	1.7025
HT450-45	1.4315	0.796	0.2495	0.71	1.813
HT750-45	1.6295	0.7405	0.273	0.653	1.7035
HT1050-45	1.5065	0.798	0.194	0.649	1.8525
UHT-60	1.7135	0.7485	0.2545	0.6985	1.5855
HT450-60	1.485	0.788	0.2485	0.7065	1.772
HT750-60	1.5225	0.6615	0.213	0.614	1.989
HT1050-60	1.5125	0.6185	0.223	0.646	2
UHT-90	1.91	0.8295	0.2005	0.5645	1.4955
HT450-90	1.8695	0.815	0.1865	0.48	1.649
HT750-90	1.905	0.76	0.2055	0.535	1.595
HT1050-90	1.761	0.8755	0.2455	0.5615	1.557

**Table 4:** The hardness data of SLM SS316L at different heat treatment temperatures.

Process	0° /HV	45° /HV	45° Ratio	60° /HV	60° Ratio	90° /HV	90° Ratio	Hardness _Ave_ /HV	σ_HV_
UHT	239.93	225.1	0.937	222.93	0.929	241.15	1.007	232.3	9.60
HT450	244.43	231.8	0.949	228.3	0.933	247.93	1.014	236.6	9.52
HT750	210.53	200.55	0.953	205.58	0.976	216.25	1.017	208.2	6.72
HT1050	195.6	190.85	0.975	186.18	0.953	205.8	1.052	194.6	8.39

Note: The ratio for each angle (45°, 60°, and 90°) is calculated by dividing the hardness value for that angle by the hardness value at 0°. A ratio close to 1 indicates minimal anisotropy, while ratios diverging from 1 reflect increasing anisotropy.

**Table 5:** The data of the tensile properties of SLM SS316L for UHT and HT specimens.

Process	Building Direction/°	UTS/MPa	Ratio (UTS)	UTS _Ave_/MPa	σ(UTS)	EL/%	Ratio (EL)	σ(EL)
UHT	0	648.61	-	631.08	16.67	14.51	-	8.98
	45	628.66	0.97			10.51	0.73	
	60	609.27	0.94			12.50	0.86	
	90	637.79	0.98			20.04	1.38	
HT450	0	655.85	-	636.61	16.03	21.25	-	7.61
	45	633.45	0.97			13.75	0.65	
	60	617.13	0.94			13.95	0.66	
	90	640.03	0.98			21.67	1.02	
HT750	0	623.15	-	612.59	14.65	22.15	-	4.33
	45	614.71	0.99			20.44	0.92	
	60	591.3	0.95			20.12	0.91	
	90	621.22	0.99			25.57	1.15	
HT1050	0	593.04	-	585.35	14.93	25.40	-	4.97
	45	582.07	0.98			20.75	0.82	
	60	565.98	0.95			23.75	0.93	
	90	600.31	1.01			27.50	1.08	

Note: rhe ratio compares the mechanical properties (UTS and EL) at different building directions (45°, 60°, and 90°) to the 0° direction, which is used as the reference.

## Data Availability

The original contributions presented in this study are included in the article. Further inquiries can be directed to the corresponding authors.
